# Data for a comparative proteomic analysis of chloroplast biogenesis (*clb*) mutants

**DOI:** 10.1016/j.dib.2014.07.001

**Published:** 2014-08-12

**Authors:** L.A. de Luna-Valdez, A.G. Martínez-Batallar, M. Hernández-Ortiz, S. Encarnación-Guevara, M. Ramos-Vega, J.S. López-Bucio, P. León, A.A. Guevara-García

**Affiliations:** a Instituto de Biotecnología, Universidad Nacional Autónoma de México, Apartado Postal 510-3, 62214 Cuernavaca, Morelos, México; b Centro de Ciencias Genómicas, Universidad Nacional Autónoma de México, Av. Universidad 565, Chamilpa, 62210 Cuernavaca, Morelos, Mexico

**Keywords:** Chloroplast development, Comparative proteomics, clb mutants, *Arabidopsis thaliana*

## Abstract

This data article contains data related to the research article titled **Proteomic analysis of chloroplast biogenesis (clb) mutants uncovers novel proteins potentially involved in the development of*****Arabidopsis thaliana*****chloroplasts** (de Luna-Valdez et al., 2014) [1]. This research article describes the 2-D PAGE-based proteomic analysis of wild-type and four mutant lines (*cla1-1*, *clb2*, *clb5* and *clb19*) affected in the development of *Arabidopsis thaliana* chloroplasts. The report concludes with the discovery of three proteins potentially involved in chloroplast biogenesis. The information presented here represent the tables and figures that detail the processing of the raw data obtained from the image analysis of the 2-D PAGE gels.

Specifications table

**Table:** 

Subject area	Biology
More specific subject area	*Plant proteomics*
Type of data	*Tables and fiures*
How data was acquired	***Electron microscopy***: *Images were extracted from*[2,3,4]
	***2-D PAGE and image analysis***: *GS-800 densitometer* (*Bio-Rad Hercules, CA, EUA*); *image analysis software PD-Quest 8.0.1* (*Bio-Rad Hercules, CA, EUA*)
	***Mass Spectrometry***: Matrix-Assisted Laser Desorption/Ionization-Time of Flight; Autoflex, Bruker Daltonics, Billerica, MA, USA
Data format	*Processed.*
Experimental factors	*No pretreatment of samples was performed.*
Experimental features	*Total protein was extracted from mutant and wild-type plants by triplicate. 2-D PAGE gel images were generated and compared in order to discover reliable (T-test P*<*0.01) spots with abundance shift of at least* ±*2-fold.*
Data source location	*NA.*

**Value of the data**

• The data further validate the information presented in de Luna-Valdez et al. (2014) [1].
• The data present alternative ways of visualizing the abundance of the proteins under study.
• The data provide specifics on the biochemical processes affected in all the analyzed clb mutants.

**Direct link to deposited data in public repository**

The data is directly available in this article and related to de Luna-Valdez et al. (2014) [1].

## 1 Experimental design

Total protein was extracted from 16-days old mutant and 8-days old wild-type plants by triplicate. 2-D PAGE gel images were generated and compared in order to discover reliable (*T*-test *P*<0.01) spots with abundance shift of at least ±2-fold. Protein identification was performed using MALDI-TOF Mass spectrometry.

## 2 Material and methods [1]

### 2.1 Plant material and growth conditions

*Arabidopsis thaliana* heterozygous mutant lines corresponding to *cla1-1* (At4g15560) [2], *clb2* (At3g11945) [3], *clb19* (At1g05750) [4], *clb5* (At3g04870) [3,5], *emb1241* (SALK_045238), *pbp1* (SAIL_773_D06), and *atrabe1b* (SALK_069644) were used in this study (Fig. S1, S2). Seeds were surface-sterilized using solutions of 100% C_2_H_6_O and 1% NaClO, then cultured on 0.5X Murashige & Skoog media supplemented with 0.05 g/l 2-(N-morpholino)ethanosulfonic acid, 0.5 g/l sucrose, 100 mg/l myo-inositol, 1 mg/l nicotinic acid, 1 mg/l pyridoxine-HCl, 10 mg/l thiamine-HCl, and 8 g/l phyto agar. Seedlings from the four mutant lines that presented the wild-type phenotype and the first pair of true leaves were harvested after 8 days of culture. These were then pooled for processing as the wild-type protein samples used in this study. In order to minimize the effect of using plants in different developmental stages (detection of development-related proteins), pigment-deficient plants were collected after 16 days of culture; at this time, all the seedlings display at least the first pair of true leaves. Three biologically independent seedling batches were generated for further processing.

### 2.2 Extraction and quantification of total protein

Total protein extracts were prepared according to the phenol extraction protocol reported by Hurkman and Tanaka [6]; the adjustments made to the original protocol are described here. Briefly, the starting plant material was 1 g of mutant or wild-type seedlings grown in vitro on GM medium, seedlings were ground in a mortar using liquid nitrogen and re-suspended in extraction buffer (0.7 M sucrose; 0.5 M Tris; 30 mM HCL; 50 mM EDTA; 0.1 MKCl, 12 mg/ml PVPP (Polyvinylpolypyrrolidone) and 2% ⋄-mercaptoethanol). An equal volume of water-saturated phenol was added followed by centrifugation (6000*g* for 10 min) to separate the phases. The phenol phase was re-extracted with one volume of extraction buffer then precipitated with 5 volumes of 0.1 M ammonium acetate in methanol at −20 °C overnight. The protein precipitate was washed three times with 0.1 M ammonium acetate in methanol and once with 80% acetone at −20 °C. The pellets were air dried under vacuum and re-suspended in lysis buffer (8 M Urea; 2 M Thiourea, 4% (w/v) CHAPS; 2% ampholines (1.5% pH range 5–7 and 0.5% pH range 3–10) and 60 mM DTT). Determination of protein concentration in the extracts was determined by colorimetric assays as reported by Encarnación et al. (2005) [7].

### 2.3 2-D PAGE and protein visualization

500 μg (analytical gels) or 750 μg (preparative gels) of total protein extracts were separated in 12% acrylamide gels under denaturating conditions. The first dimension was run using ampholytes in the range of pH 3–10 and enriched in pH 4–8. After 2-D electrophoresis, gels were fixed and stained using colloidal Coomassie brilliant blue, following [7]. The stained gels were digitalized using a GS-800 densitometer (Bio-Rad Hercules, CA, EUA) and the image analysis software PD-Quest 8.0.1 (Bio-Rad Hercules, CA, EUA) (Fig. S1).

### 2.4 In silico processing of gel images

Images from 2-D gels of three biologically independent protein extracts from mutants (*cla1-1*, *clb2*, *clb5*, and *clb19*) and wild-type plants were generated and processed using the PD-Quest 8.0.1 software (Bio-Rad, Hercules CA) (Fig. S1). Protein spots in all replicates were detected automatically by the software, and the detection was then improved by the manual addition of missing spots and the removal of erroneously detected spots. Normalization of gel images was performed using the local regression model normalization method provided by PD-Quest software. Furthermore, in order to properly compare the samples, the gel images were adjusted to fit a common distortion model; this was done by matching spots that were common to all the gel images. The gel images from the different protein samples were compared to each other in order to generate a robust data set containing all the spots represented in the samples with 98% statistical confidence (*P*<0.01) in a Student׳s *t*-test. Finally, the protein spots in the statistical data set displaying ±2-fold abundance change were selected as candidates for the MS analysis (Table S1).

### 2.5 MALDI-TOF mass spectrometry and protein identification

The selected protein spots were manually excised from the preparative gels. The samples were alkylated, reduced, and trypsin-digested prior to their elution and MS analysis. Samples of digested protein spots were automatically transferred to MALDI-TOF (Matrix-Assisted Laser Desorption/Ionization-Time of Flight; Autoflex, Bruker Daltonics, Billerica, MA, USA) using Proteineer SP and SPII systems (software SPcontrol 3.1.48.0v; Bruker Daltonics, Breme, Germany). The Bruker Daltonics Autoflex system was operated in the delayed extraction and reflectron mode, and the resolution threshold was set to a signal-to-noise ratio of 1500. The specific protocols can be accessed in [7]. The *m*/*z* spectra were searched against the Arabidopsis thaliana NCBInr (http://www.ncbi.nlm.nih.gov/guide/proteins/), SwissProt (http://www.isb-sib.ch/), and IPI (http://www.ebi.ac.uk/IPI/IPIarabidopsis.html) databases, using the Mascot (http://www.matrixscience.com) and Profound (http://prowl.rockefeller.edu) search engines. The Mascot engine was used to query NCBInr and SwissProt databases, while Profound was used to query NCBInr and IPI databases; both search engines were operated using a mass tolerance of 200 ppm, with cysteine carbamidomethylation as the constant modification and methionine oxidation as the variable modification. The significance threshold was set to *P*<0.05 for the Mascot search.

### 2.6 In silico analysis of the identified proteins

Functional clustering of the identified proteins was performed using the Functional annotation tool available at DAVID (http://david.abcc.ncifcrf.gov/home.jsp) [8], and clustering was carried out using the annotations available at the Protein Information Resource (http://pir.georgetown.edu/pirwww/index.shtml) and Gene Ontology (http://www.geneontology.org/) databases. Stringency of the classification was set on medium and the rest of the options were set as default (Fig. S3). Reconstruction of metabolic pathways was achieved using the metabolism overview pathways in the MapMan 3.5.1 software (http://mapman.gabipd.org/web/guest/mapman) with the Ath_AGI_TAIR9_Jan2010 mappings. MapMan was fed an array of data containing, for each protein, the log_2_ of the ratio of the detected abundance in each mutant over that registered in wild-type plants (Table S2).

## Footnotes

Appendix A Supplementary data associated with this article can be found in the online version at doi:10.1016/j.dib.2014.07.001.
