# Supplementary material for: Data for a comparative proteomic analysis of chloroplast biogenesis (clb) mutants
Source: Data Brief. 2014 Aug 12;1:15–8. doi: 10.1016/j.dib.2014.07.001 (PMC4459866; doi:10.1016/j.dib.2014.07.001)
Supplement: Supplementary file 1 — Supplementary Data [file mmc1.docx]

Proteomic analysis of chloroplast biogenesis (*clb*) mutants uncovers novel proteins potentially involved in the development of *Arabidopsis thaliana* chloroplasts.

de Luna-Valdez, L. A., Martínez-Batallar, A. G., Hernández-Ortiz. M., Encarnación-Guevara. S., Ramos-Vega, M., López-Bucio, J. S. , León, P. and Guevara-García, A. A.^[[1]](#footnote-1)^

**SUPPLEMENTARY MATERIAL**

**Supplemental Tables**

**Table S1.** Classification of up-regulated, down-regulated, and inconsistently abundant proteins. [Attached Excel File Supplementary Table SI.xlsx].

**Table S2.** Protein content of the bins shown in Fig. 5. [Attached Excel File Supplementary Table SII.xlsx]

**Supplemental Figure Legends**

**Figure S1. 2-D PAGE Replicates.** Images of all 2-D gels made from the three biologically independent replicates (R1-3) of protein extracts from wild-type plants (Wt) and *cla1-1*, *clb2*, *clb5,* and *clb19* mutants. The mean coefficient of variation (CV) of each replicate group is displayed in parentheses.

**Figure S2.** Plastid ultrastructure from wild-type plants (Wt) and *cla1-1*, *clb2*, *clb5,* and *clb19* mutants.

*Gutierrez-Nava, L. M., Gillmor, S. C., Jimenez, L. F., Guevara-García, A., and León, P. (2004). *Chloroplast biogenesis* (*CLB*) genes act cell and non-cell autonomously in early chloroplast development. Plant Physiology, 135: 471-482 (<http://www.plantphysiol.org> "Copyright American Society of Plant Biologists”)

**Mandel, M. A., Feldmann, K. A., Herrera-Estrella, L., Rocha-Sosa, M. and León P. 1996. CLA1, a novel gene required for chloroplast development, is highly conserved in evolution. Plant J. 9: 649-658. (<http://onlinelibrary.wiley.com/journal/10.1111/(ISSN)1365-313X> "Copyright Society for Experimental Biology”.

***Chateigner-Boutina, A.L., Ramos-Vega, M., Guevara-García, A., Andrés, C., Gutiérrez-Nava, M., Cantero, A., Delannoya, E., Jiménez, L.F., Lurinc, C., Small, I. and León, P. (2008) CLB19, a novel pentatricopeptide repeat protein required for editing of rpoA and clpP chloroplast transcripts in Arabidopsis. Plant J*.*, 56: 590-602. (<http://onlinelibrary.wiley.com/journal/10.1111/(ISSN)1365-313X>; "Copyright Society for Experimental Biology”.

**Figure S3.** Clusters formed by the functional annotation tool of DAVID (<http://david.abcc.ncifcrf.gov/home.jsp>) using the down-regulated set of proteins. Annotation categories used are shown to the left, clustered GO terms are underlined and colored blue; protein counts and statistical validation of each clustered term are displayed on the right side.

**Supplemental Figures**

**
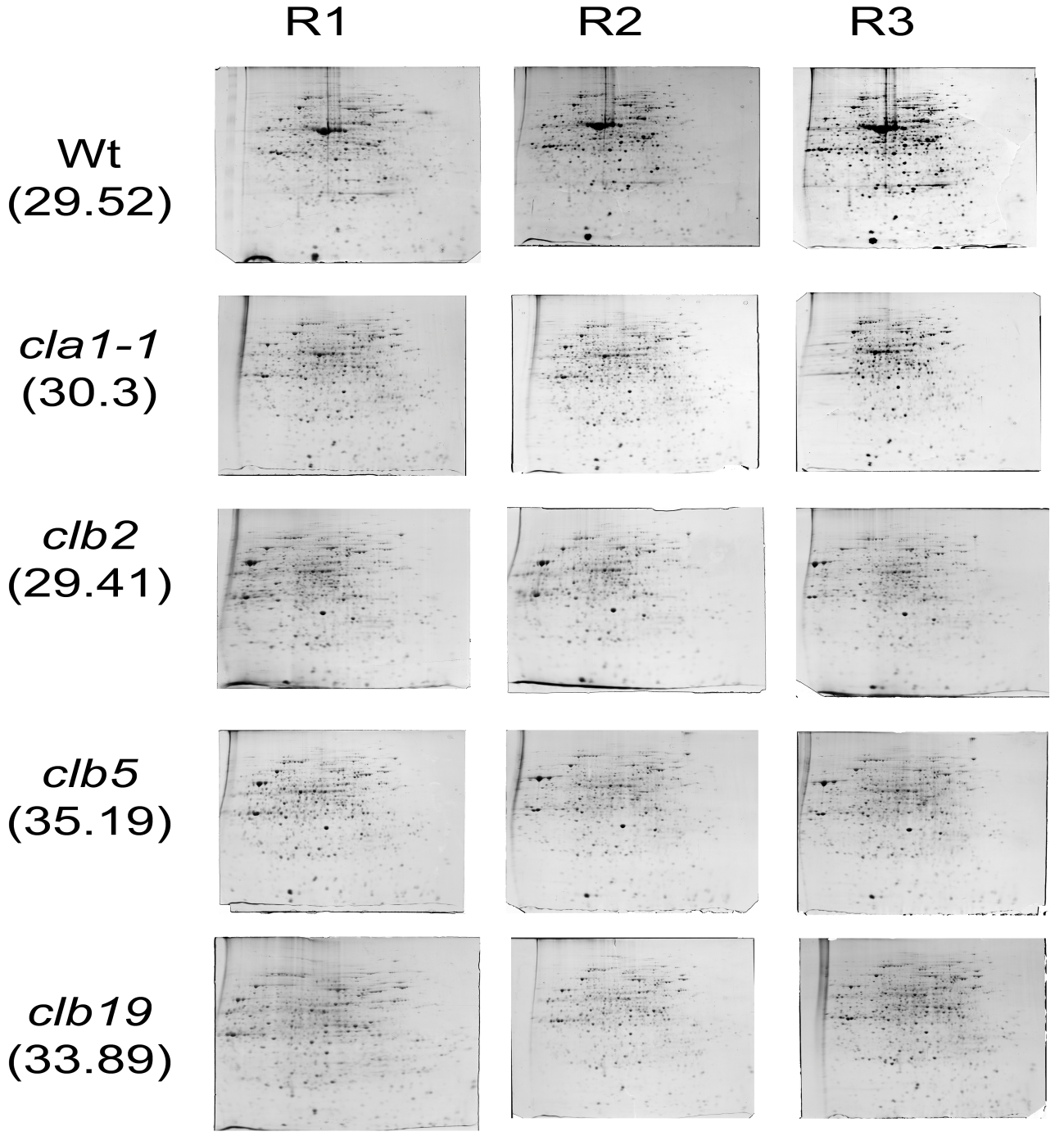
**

**Figure S1.**

**
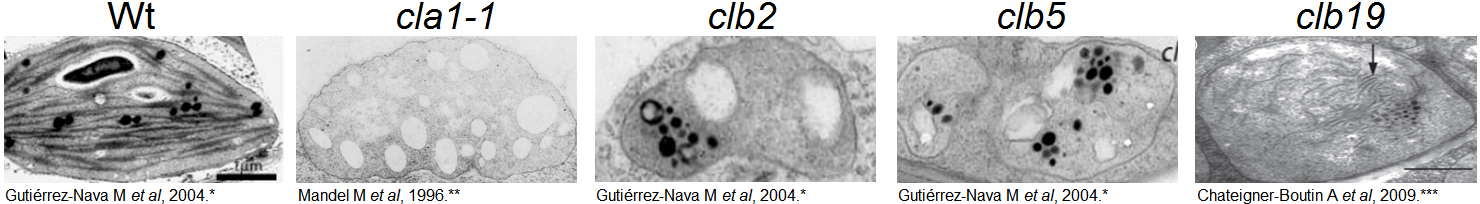
**

**Figure S2.**

**
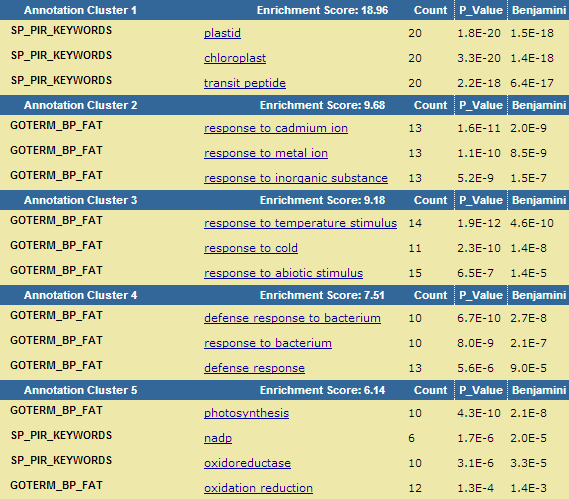
**

**Figure S3.**

1. [aguevara@ibt.unam.mx](mailto:aguevara@ibt.unam.mx) [↑](#footnote-ref-1)
